# Regulation of Opsin Gene Expression by DNA Methylation and Histone Acetylation

**DOI:** 10.3390/ijms23031408

**Published:** 2022-01-26

**Authors:** Jin Song, Julia A. VanBuskirk, Shannath L. Merbs

**Affiliations:** 1 Department of Pathology, Johns Hopkins University School of Medicine, Baltimore, MD 21287, USA; 2 Department of Ophthalmology, Johns Hopkins University School of Medicine, Baltimore, MD 21287, USA; julvanbusk@gmail.com; 3 Department of Ophthalmology and Visual Sciences, University of Maryland School of Medicine, Baltimore, MD 21287, USA

**Keywords:** opsin, DNA methylation, histone acetylation, retina

## Abstract

One important role of epigenetic regulation is controlling gene expression in development and homeostasis. However, little is known about epigenetics’ role in regulating opsin expression. Cell cultures (HEK 293, Y79, and WERI) producing different levels of opsins were treated with 5-aza-2’-deoxycytidine (5-Aza-dc) and/or sodium butyrate (SB) or suberoylanilide hydroxamic acid (SAHA) for 72 h. Global DNA methylation, site-specific methylation, and expressions of opsins were measured by LUMA assay, bisulfite pyrosequencing, and qPCR, respectively. Mouse retinal explants from wild-type P0/P1 pups were ex vivo cultured with/without 5-Aza-dc or SAHA for 6 days. The morphology of explants, DNA methylation, and expressions of opsins was examined. The drugs induced global DNA hypomethylation or increased histone acetylation in cells, including DNA hypomethylation of rhodopsin (*RHO*) and L-opsin (*OPN1LW*) and a concomitant increase in their expression. Further upregulation of *RHO* and/or *OPN1LW* in HEK 293 or WERI cells was observed with 5-Aza-dc and either SB or SAHA combination treatment. Mouse retinal explants developed normally but had drug-dependent differential DNA methylation and expression patterns of opsins. DNA methylation and histone acetylation directly regulate opsin expression both in vitro and ex vivo. The ability to manipulate opsin expression using epigenetic modifiers enables further study into the role of epigenetics in eye development and disease.

## 1. Introduction

Epigenetic regulation of the genome is important for orchestrating tissue-specific gene expression during mammalian development. DNA methylation and histone modifications are two epigenetic mechanisms affecting gene expression without changing the actual DNA sequence. DNA methylation, occurring mostly as 5′-methylcytosine (5-mC) in CpG dinucleotides, is typically associated with chromatin condensation and gene silencing [1]. About 70% of CpGs within the genome are methylated. Most unmethylated CpGs are found in CpG islands, creating a permissive chromatin environment for gene transcription [2]. Many CpG island-containing genes associated with neurogenesis show dynamic changes in methylation during development and demonstrate tissue-specific and developmentally-restricted expression patterns [3,4]. The covalent modifications of N-terminal tails of histones by acetylation, methylation, phosphorylation, or ubiquitylation also lead to changes in chromatin structure and gene expression. DNA methylation and histone modifications influence each other during mammalian development. Histone methylation is thought to help direct DNA methylation patterns, while DNA methylation can serve as a template for some histone modifications [5].

Gene expression can be modified through pharmacological manipulation of DNA methylation and histone acetylation. DNA methylation patterns are established and maintained by structurally distinct family members of DNA methyltransferase (DNMT) enzymes [6]. 5-azacytidine (5-Aza), 5-aza-2′-deoxycytidine (5-Aza-dc), 5, 6-dihydro-5-azacytidine, zebularine, procaine, and RG108 are DNMT inhibitors (DNMTi) used in research, and some have been tested in preclinical and clinical studies to treat cancer [7,8]. 5-Aza and its analogs can be incorporated into DNA, leading to blockage of cytosine methylation via covalent trapping of DNMT [9]. Histone acetylation is a dynamic process controlled by histone acetyltransferases (HAT) and histone deacetylases (HDAC; including class I, II, and III) [10]. HDAC inhibitors (HDACi) such as sodium butyrate (SB), trichostatin A (TSA), and suberoylanilide hydroxamic acid (SAHA) are also used extensively in both research and clinical trials to target histone acetylation [11,12,13,14]. HDACi increases the acetylation of histones, thereby permitting an open chromatin state associated with increased transcriptional activity [12]. Combination treatment with both a DNMTi and an HDACi appears to be more effective in modulating gene expression, although the ideal combination and timing are still being determined [15,16].

In contrast to numerous reports related to the role of epigenetic regulatory mechanisms in cancer, embryonic stem cells, and other cell types, the role of epigenetics in the development of the neural retina, including the regulation of opsin expression during the development of rods and cones, is poorly understood [3,17,18,19,20]. Human retinoblastoma cell lines Y79 and WERI, and HEK 293 (known to have some neuronal features), have differential methylation of L-opsin (*OPN1LW*) and M-opsin (*OPN1MW*), which is inversely correlated with gene expression levels [21,22]. Previous studies have shown that DNMT1-dependent DNA methylation and histone deacetylase activity are required to express critical photoreceptor genes, photoreceptor terminal differentiation, and retinal neuron survival [23,24]. In the explant culture of the murine retina, HDAC activity is required for the expression of key neuronal pro-rod transcription factors and the development of rod photoreceptor cells [23]. How these epigenetic modifications regulate opsin gene expression during the development of mouse photoreceptor cells still remains largely unknown. Bisulfite sequence analyses in vitro and ex vivo have shown that photoreceptor-specific genes have cell-specific differential DNA methylation patterns that are inversely proportional to their expression levels, suggesting that DNA methylation may play an important role in modulating photoreceptor gene expression in the developing mammalian retina [22]. In this study, we further investigate the possible role of DNA methylation and histone acetylation in the regulation of opsin gene expression through pharmacological manipulation of these processes, both in vitro and ex vivo.

## 2. Results

### 2.1. Treatment of Tissue Culture Cells with 5-Aza-dc or HDACi Induces a Global DNA Hypomethylation or Increased Histone Acetylation

**Figure 1:**
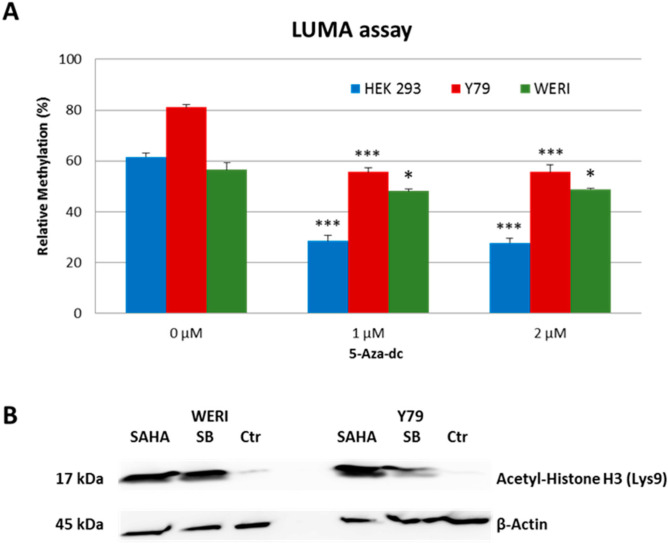
Effect of 5-Aza-dc or HDACi on DNA methylation or histone acetylation in cell culture. (**A**) 5-Aza-dc treatment of HEK 293, Y79, and WERI cells results in a global DNA hypomethylation in all three cell lines. Student’s *t*-test: *, *p* < 0.05 and ***, *p* < 0.001; treated vs untreated cells. The relative methylation (%) is the average of triplicates ± SD. (**B**) Western blot of WERI and Y79 cells treated with HDACi shows increased acetyl-Histone H3 (Lys9). β-actin serves as a loading control.

Using the LUMA assay to measure overall genome-wide methylation, 5-Aza-dc treatment of HEK 293, Y79, and WERI cells for 3 days resulted in global DNA hypomethylation in all three cell lines (Figure 1A). After Y79 and WERI cells were treated with HDACi for 3 days, increased histone acetylation was observed by Western blot for both SB and SAHA treatment (Figure 1B).

### 2.2. 5-Aza-dc and/or HDACi Treatment Results in DNA Hypomethylation of RHO and OPN1LW and Increased Expression in Tissue Culture Cells

**Figure 2:**
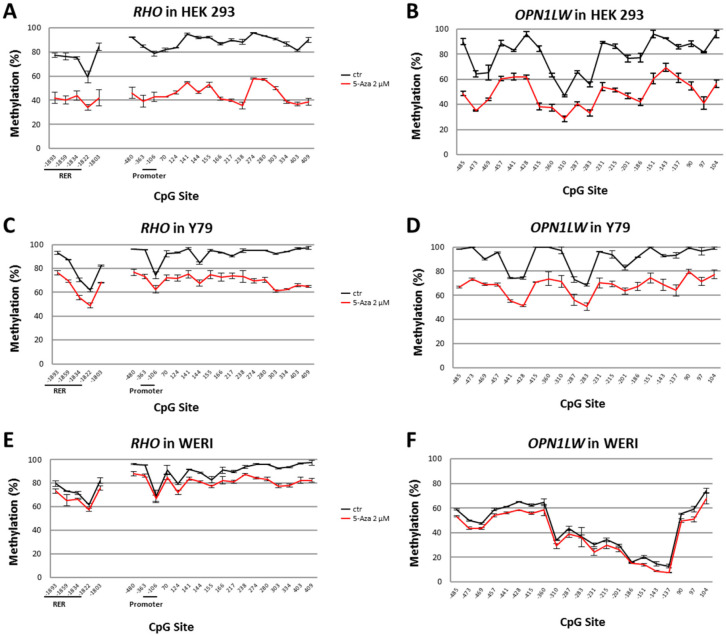
5-Aza-dc induce DNA hypomethylation of *RHO* and *OPN1LW* in tissue culture cells. (**A**,**B**) HEK 293. (**C**,**D**) Y79 (**E**,**F**) WERI. 2 µM 5-Aza-dc treatment significantly decreases DNA methylation at CpG sites around the TSS and RER of *RHO* and the TSS of *OPN1LW*. Student’s *t*-test: *p* < 0.0001 for HEK 293 and Y79, *p* < 0.002 for WERI. The methylation (%) is the average of triplicates ± SD.

Bisulfite pyrosequencing and qPCR assays were performed to determine the effect of drug treatment on DNA methylation and mRNA expression of representative rod and cone photoreceptor-specific genes (*RHO* and *OPN1LW*) in HEK 293, Y79, and WERI cells. The methylation level of 17 CpGs within 900 bp around the transcription start site (TSS) of *RHO*, 21 CpGs within 600 bp around the TSS of *OPN1LW*, and 5 CpGs located in the 100 bp rhodopsin enhancer region (RER) located 1.8–1.9 kb upstream of the TSS of *RHO* [25] were measured (Figure 2). Compared with untreated cells, treatment with both doses of 5-Aza-dc (1 or 2 µM) resulted in a significant decrease in DNA methylation around the TSS and RER of *RHO* and the TSS of *OPN1LW* in all three cell lines (*p* < 0.0001 for HEK 293 and Y79, *p* < 0.002 for WERI; only 2 µM 5 Aza-dc treatment shown in Figure 2). Similar DNA hypomethylation levels were seen with 5-Aza-dc combined with SB or SAHA treatments (data not shown). This effect was not seen for SB or SAHA-only treatments (data not shown). 

**Figure 3:**
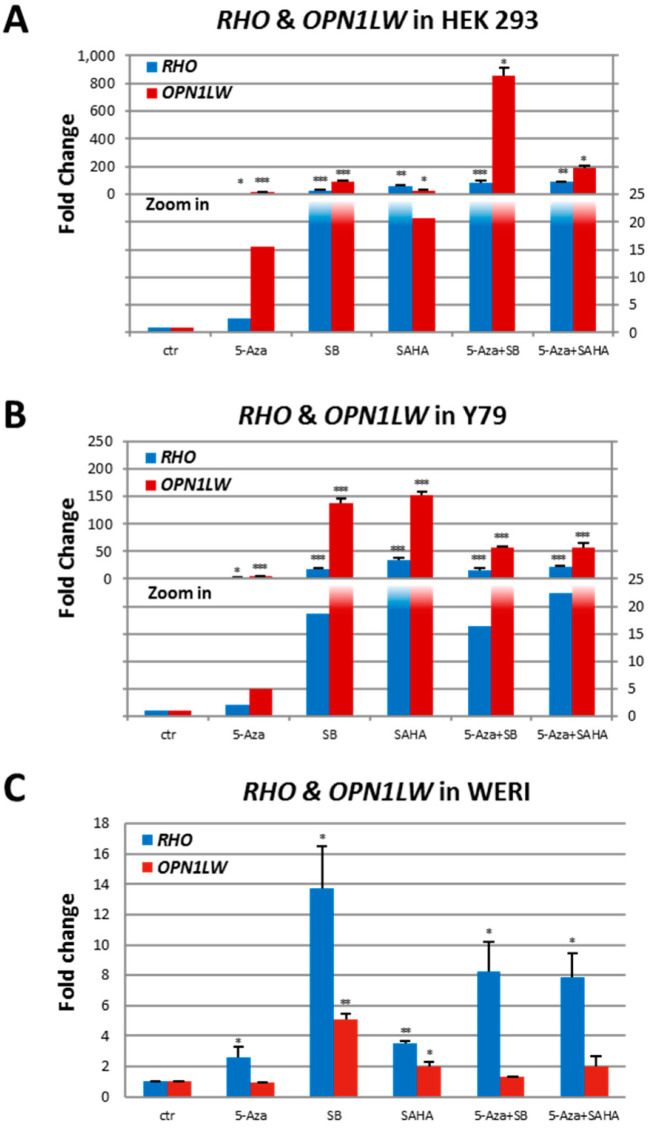
5-Aza-dc and/or HDACi enhance *RHO* and *OPN1LW* gene transcription in tissue culture cells. (**A**) HEK 293. (**B**) Y79. (**C**) WERI. Treatment with 5-Aza-dc (2 µM) or HDACi (SB, 2 mM or SAHA, 3 µM) of HEK 293, Y79, and WERI cells results in a significant increase of *RHO* or *OPN1LW* expression in all cases except 5-Aza-dc on *OPN1LW* in WERI cells. Treatment with both 5-Aza-dc and an HDACi further upregulates the expression of *RHO* and *OPN1LW* in HEK 293 cells and *RHO* in WERI cells. Student’s *t*-test: drug versus control, *, *p* < 0.05; **, *p* < 0.01; ***, *p* < 0.001. Normalized to *HPRT1*, *HMBS* & *GAPDH*. The fold change is the average of triplicates ± SD.

Furthermore, 5-Aza-dc treatment resulted in a significant increase of *RHO* mRNA in all three cell types (Figure 3A–C) and of *OPN1LW* mRNA in HEK 293 and Y79 cells (Figure 3A,B) compared with the untreated cells. SB or SAHA alone also significantly upregulated mRNA expression of *RHO* or *OPN1LW* in all three cell types (Figure 3A–C). 5-Aza-dc and SB or SAHA combination treatments demonstrated an increased effect in upregulating mRNA expression of *RHO* and *OPN1LW* in HEK 293 (either combination treatment versus their respective single drug treatment; *p* < 0.001 for all comparisons, except 5 Aza-dc and SAHA versus SAHA at *p* < 0.01; Figure 3A) and mRNA expression of *RHO* in WERI cells (5-Aza-dc and SAHA combination treatment versus their respective single drug treatment; *p* < 0.05; Figure 3C).

### 2.3. Role of DNA Methylation and Histone Deacetylase Activity on Expressions of Opsin Genes during the Development of Mouse Photoreceptor Cells

**Figure 4:**
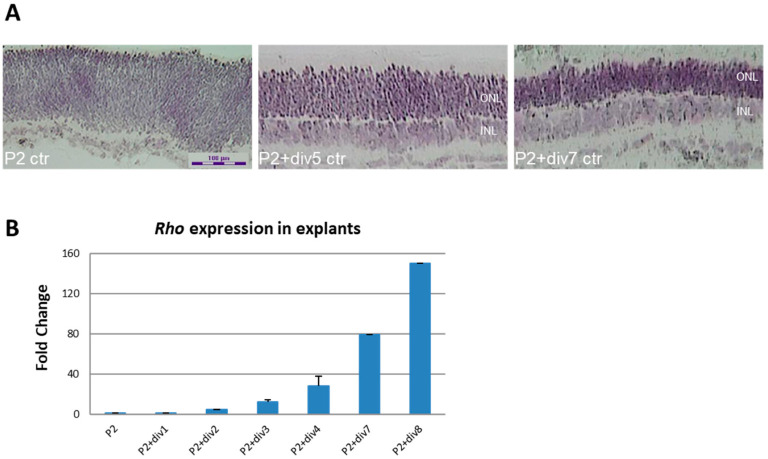
Morphologic differentiation and expression of *Rho* in mouse retinal explant ex vivo culture. (**A**) Morphologic differentiation in culture results in the formation of well-defined inner (INL) and outer nuclear layers (ONL), inner and outer plexiform layers, and ganglion cell layer as seen by crystal violet staining (20x magnification). (**B**) expression of mouse *Rho* increased exponentially from P2+div3 to P2+div8. Normalized to mouse *Cyclophilin*. The fold change is the average of triplicates ± SD, relative abundance to P2.

As previously reported [26,27,28], we were able to culture mouse retinal explant ex vivo and characterize the temporal expression patterns of mouse *Rho* in retinal explants without drug treatment (Figure 4). Mouse retinal explants obtained from wild-type P2 pups that were ex vivo cultured until P2+div5 and P2+div7 underwent morphologic differentiation in culture, resulting in the formation of well-defined inner (INL) and outer nuclear layers (ONL), inner and outer plexiform layers, and a ganglion cell layer (Figure 4A). Expression of mouse *Rho* at P2, P2+div1, P2+div2, P2+div3, P2+div4, P2+div7, and P2+div8 increased exponentially from P2+div3 to P2+div8 (Figure 4B).

**Figure 5:**
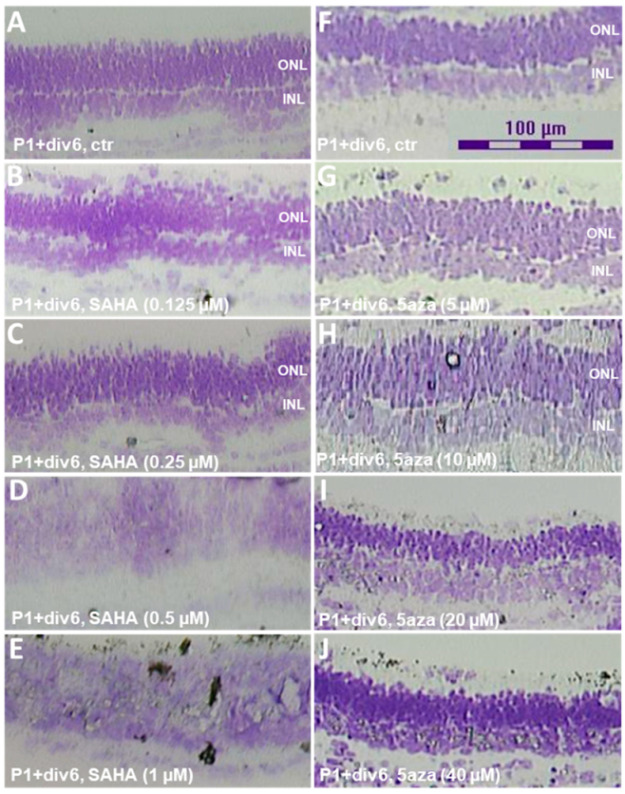
Histological analysis of dose-dependent effects of 5-Aza-dc or SAHA in mouse retinal explants. (**A**–**C**) Proper lamination and differentiation of the mouse retinal retinas in concentrations up to 0.25 µM of SAHA or (**F**–**I**) 10 µM of 5-Aza-dc. (**D**,**E**,**J**) Abnormal retinal differentiation at higher drug concentrations. Crystal violet staining, 20× magnification.

To explore the effect of DNA methylation and histone acetylation on retinal cell viability and retinal development [29], P1 mouse retinal explants were first ex vivo cultured under the different doses of 5-Aza-dc or SAHA until P1+div6. Histological analysis showed obvious proper lamination and differentiation of the retinas up to 20 µM of 5-Aza-dc or 0.25 µM of SAHA treatments (Figure 5). 5 µM of 5-Aza-dc or 0.25 µM of SAHA was used for the downstream time-course experiments. 

**Figure 6:**
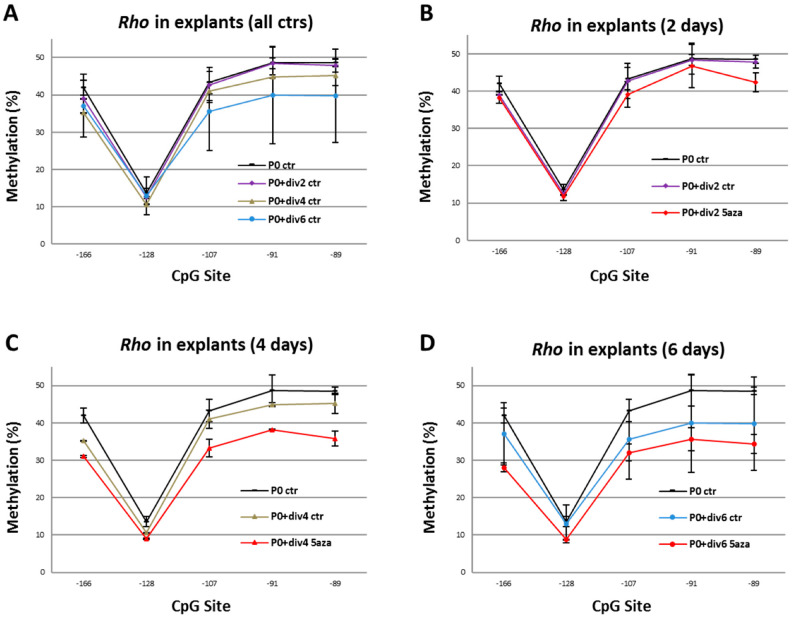
Pyrosequencing analysis of mouse *Rho* proximal promoter region in retinal explants grown in 5-Aza-dc. (**A**) Mouse retinal explants ex vivo controls (ctr) demonstrate a stepwise decrease in DNA methylation with increased time in culture (*p* = 0.01). (**B**–**D**) At each respective timepoint, culture in the presence of 5-Aza-dc (5 µM) reduced the methylation level compared to untreated explants (*p* < 0.01 at P0+div4 and *p* < 0.05 at P0+div6). Student’s *t*-test. The methylation (%) is the average of triplicates ± SD.

P0 mouse retinal explants were ex vivo cultured in the presence or absence of 5 µM of 5-Aza-dc or 0.25 µM of SAHA until P0, P0+div2, P0+div4, and P0+div6 and analyzed by pyrosequencing and qPCR assays. In the untreated explants, a stepwise decreased methylation level with a significant difference of methylation level between P0+div6 and P0 (*p* = 0.01; Figure 6A) was observed, especially for the proximal 3 CpGs, which corresponded to the expression pattern of mouse *Rho* during the development of mouse photoreceptor cells (Figure 4B). In contrast, 5-Aza-dc-treated retinal explants ex vivo cultured demonstrated much lower methylation levels compared with untreated retinas at all time points (Figure 6B–D). There was a significant difference in methylation level between 5-Aza-dc treated explants and untreated ones at P0+div4 (*p* < 0.01; Figure 6C) and P0+div6 (*p* < 0.05; Figure 6D).

**Figure 7:**
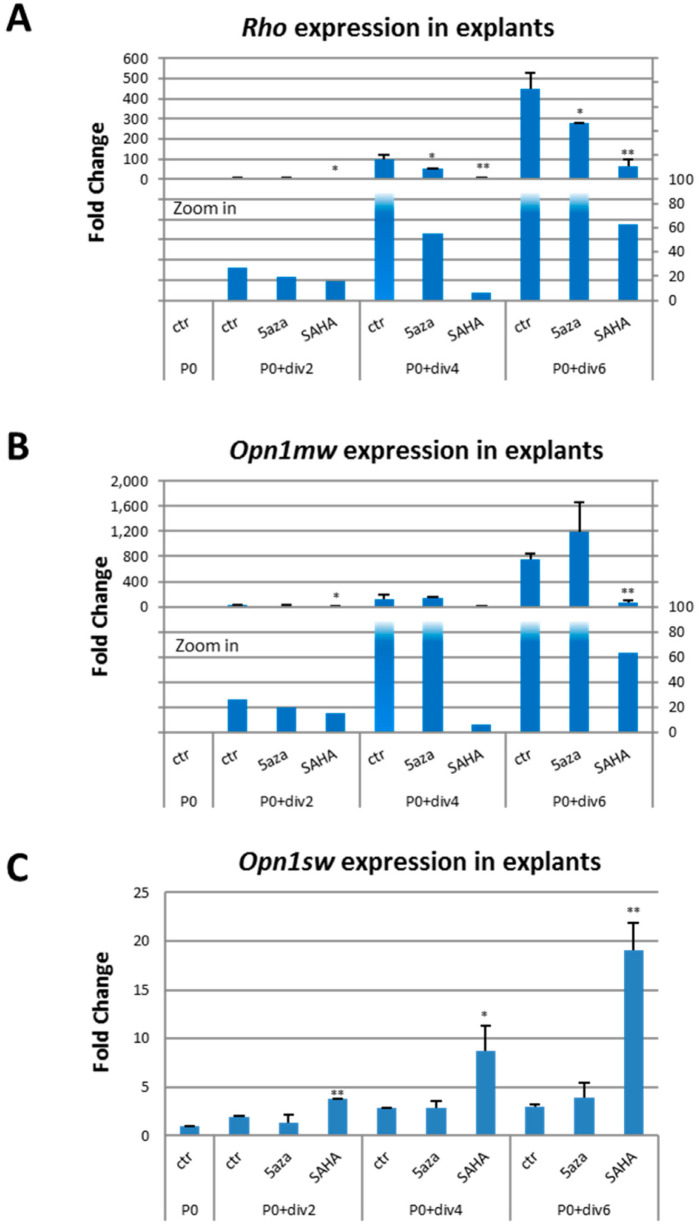
Effect of DNMTi and HDACi on the expression of opsin genes during murine retinal development. (**A**–**C**) The expression levels of *Rho*, *Opn1mw*, *Opn1sw* increase in untreated ex vivo cultured explants (ANOVA: *p* = 0.001 for *Rho*; *p* = 0.0007 for *Opn1mw*; *p* < 0.0001 for *Opn1sw*). Expression level of *Rho* increase over time in culture with drug treatment (ANOVA: *p* < 0.0001 for 5-Aza-dc and *p* = 0.09 for SAHA), both 5-Aza-dc and SAHA treatment significantly decreased mouse *Rho* expression in postnatal retinal explants ex vivo cultured relative to no drug treatment. In addition, SAHA significantly increased mouse *Opn1sw* but decreased *Opn1mw* expression level. Normalized to mouse *Cyclophilin*. Student’s *t*-test: drug versus ctr at the same time point; *, *p* ≤ 0.05; **, *p* ≤ 0.01. The fold change is the average of triplicates ± SD, relative abundance to P0 ctr.

qPCR analysis of changes in *Rho*, *Opn1mw* or *Opn1sw* expression in P0 retinal explants ex vivo cultured showed a steady increase in the expression levels of all three mouse opsin genes in untreated explants (ANOVA: *p* = 0.001 for *Rho*, *p* = 0.0007 for *Opn1mw*, *p* < 0.0001 for *Opn1sw;* Figure 7A–C). SAHA significantly increased *Opn1sw* expression (Figure 7C) but decreased *Rho* (Figure 7A) and *Opn1mw* expression (Figure 7B) compared to untreated explants. 5-Aza-dc significantly decreased mouse *Rho* (Figure 7A) expression level in 5-Aza-dc treated explants compared with untreated ones, while it had no significant effect on *Opn1mw* (Figure 7B) and *Opn1sw* (Figure 7C) expression levels. In contrast to the in vitro observation in human adult tissue culture cells (HEK 2933, Y79, and WERI), in which both 5-Aza-dc and SAHA treatments significantly increased *RHO* expression, in developing postnatal retinal explants, 5-Aza-dc and SAHA treatments decreased mouse *Rho* expression at each time point relative to no drug treatment. However, the expression level of *Rho* did steadily increase over time in culture with drug treatments (ANOVA: *p* < 0.0001 for 5-Aza-dc and *p* = 0.09 for SAHA; Figure 7A), such that drug treatment essentially shifted the time-course expression curve to the left and earlier time points.

## 3. Discussion

The coordinated and precise regulation of opsin expression is crucial for retinal development and homeostasis. Under- or over-expression of certain opsins can cause abnormalities, dysfunction and/or death of retinal photoreceptors, eventually resulting in various retinal degenerations. Recent evidence demonstrates that epigenetic regulation, including DNA methylation and histone modification, is involved in photoreceptor cell fate specification and terminal differentiation during retinal development as well as in homeostasis in the adult retina [3,20,30]. Using DNMTi and HDACi, we were able to pharmacologically manipulate rod and cone photoreceptor-specific gene expression both in vitro and ex vivo, suggesting that DNA methylation and histone acetylation are directly involved in regulating opsin expression. Using DNMTi and HDACi in terminally differentiated retinoblastoma cells (Y79 and WERI) and immortalized cells of non-retinal origin (HEK 293), we found upregulation of opsins by drug treatment in both expressing (i.e., *OPN1LW* in WEI and Y79) and non-expressing (i.e., *RHO* and *OPN1LW* in HEK 293) cell lines, consistent with previous studies [31,32,33,34]. In contrast, postnatal retinal explants cultured ex vivo with 5-Aza-dc showed decreased mouse *Rho* expression, essentially shifting the *Rho* expression time-course curve to the left and an earlier time point. These differing drug effects between terminally differentiated adult cells and developing retina cells indicate that these epigenetic modifications’ roles may differ in the adult and developing retina.

We have a very limited understanding of how sequential changes in epigenetic state occur in photoreceptors during retinal development and how epigenetic mechanisms are involved in the different steps of photoreceptor development, such as the proliferation of retinal progenitor cells, photoreceptor cell fate specification, expression of photoreceptor-specific genes and terminal differentiation. Interestingly, in retinal explants, SAHA significantly upregulated mouse *Opn1sw* expression while downregulating *Rho* and *Opn1mw* expression. In the mouse, cones are born on about ~E11, with *Opn1sw* expression beginning at ~E18 and *Opn1mw* expression beginning at ~P6. Rods are generated both pre-and postnatally from ~E12 to P10, and like *Opn1mw*, the expression of *Rho* begins postnatally at ~P2. Therefore, SAHA upregulated *Opn1sw*, which was already being expressed at the time of explant harvest, and downregulated *Opn1mw* and *Rho*, the expression of which had not begun by the time of explantation. Previous experiments have shown that P2 mouse retinal explants cultured with or without TSA, another HDACi, for 8 days resulted in increased cell death, reduction in proliferation, a complete loss of *Rho*-positive photoreceptors, and an increase in *Chx10*-positive bipolar cells, indicating a cell fate switch due to the HDAC inhibition at the early stage of photoreceptor development [23]. This study demonstrated a significant delay in *Rho* expression in postnatal retinal explants ex vivo cultured through the more sensitive QPCR analysis, which confirmed the previous observations [23]. It also should be noted that there was a slight difference in the timing of explanation (P0 vs. P2), the different HDACis used, and/or drug toxicity in these studies. In contrast to cell death and reduced proliferation [23], we observed the appropriate morphologic differentiation of the explant ex vivo cultured and temporal expression of opsins suggesting that the effect of SAHA on opsin expression was not an effect of cytotoxicity.

One might have expected *Rho* expression to actually increase as it became less methylated; in the adult retina, *Rho* is methylated in non-expressing cells from the INL and unmethylated in expressing cells from the ONL [22]. Because 5-Aza-dc treatment reduced *Rho* expression in the developing mouse retina, maintenance of a certain level of DNA methylation of *Rho* or other genes prior to expression of *Rho* must be important for the subsequent development of rods and expression of *Rho*. Consistent with this explanation is the high level of *Dnmt* expression observed in the early stages of retinal differentiation [35] and the hindered photoreceptor differentiation with *Dnmt1* knockdown [24]. Our current understanding of photoreceptor differentiation is mainly based on research on mice and other organisms. However, some molecules expressed in the human and mouse retina may have different molecular control mechanisms, resulting in temporal and spatial differences between species. The 3D retina derived from human embryonic stem cells (hESC) has recently been developed as an in vitro model system that can recapitulate the time course of retinal differentiation in vivo while demonstrating the commonalities and differences between humans and mice and provide a better platform for studying the pathways of human photoreceptor development [36].

## 4. Materials and Methods

### 4.1. Cell Culture and Drug Treatment

Two human retinoblastoma cell lines (WERI-Rb1 and Y79) and the non-neuronal human embryonic kidney cell line (HEK 293) were purchased from ATCC (Manassas, VA, USA). Cells were maintained in DMEM (WERI and HEK 293) or RPMI-1640 (Y79) medium supplemented with 10% (*v*/*v*) heat-inactivated FBS. Media, supplements, and antibiotics were purchased from Invitrogen (Carlsbad, CA, USA). Drugs include 5-Aza-dc (A3656) and 2 structurally different inhibitors for class I/II HDACs (SB, B5887; SAHA, SML0061) purchased from Sigma-Aldrich (Saint Louis, MO, USA). Both 5-Aza-dc and SB were dissolved in water, while SAHA was dissolved in DMSO. In triplicate, cells were treated with 5-Aza-dc (1 or 2 µM) or SB (2 mM) or SAHA (3 µM) or combination(s) for 72 h (the fresh drug was added every other day).

### 4.2. Animals

The experiments were performed in accordance with the ARVO Statement for the Use of Animals in Ophthalmic and Vision Research, with the approval of the Johns Hopkins University Institutional Animal Care and Use Committee (protocol MO12M217). C57BL/6J wild-type mice (The Jackson Laboratory, Bar Harbor, ME) were used. Day of birth was considered postnatal day 0 (P0).

### 4.3. Ex Vivo Retinal Explant Cultures

C57BL/6J wild-type mice at P0, P1, or P2 were decapitated. The eyes were enucleated and incubated in FBS-free DMEM medium (Sigma-Aldrich, Saint Louis, MO, USA) supplemented with 1% penicillin-streptomycin (Invitrogen, Carlsbad, CA, USA) and 0.1% fungizone® antimycotic (Invitrogen) at 37 °C for 15 min. The eyes were dissected aseptically in a Petri dish containing 10 mL of FBS-free DMEM medium as described before [28], with some modifications. Briefly, the anterior segment, lens, vitreous body, sclera, and choroid were carefully removed, and the retina was cut perpendicular to its edges in half. Subsequently, the retina was transferred with a wide-bore P1000 pipette tip and flat-mounted onto a 13 mm (0.2 µM pore-size) nucleopore polycarbonate filter (Whatman, Maidstone, UK) with the photoreceptor layer facing up. The filter was then put into a 6-well culture plate floating on the top of the medium, and the retinal explant with RPE was cultured at 37 °C for up to 8 days in 2 mL of DMEM medium containing 10% FBS with or without drug. Half of the medium was exchanged every two days. Dose-dependent experiments of 5-Aza-dc (0, 5, 10, 20 and 40 µM) or SAHA (0, 0.125, 0.25, 0.5 and 1 µM) treatments using P1+6 days in vitro (div6) explants, followed by time-course experiments of 5-Aza-dc (5 µM) or SAHA (0.25 µM) treatments with P0, P0+div2, P0+div4 and P0+div6 explants were performed. To check the morphologic differentiation of the ex vivo cultured mouse retinal explants, the explants were embedded in cryopreservation molds containing a 2:1 mixture of 25% sucrose to OCT (Tissue-Teck, Fisher Scientific, Waltham, MA, USA) for H&E or crystal violet staining. Both explants derived from the same retina were combined as a single data entry and used for downstream experiments.

### 4.4. LUMA and Bisulfite Pyrosequencing Assays

Genomic DNA was isolated from cell pellets or retinal explants using either DNeasy Blood & Tissue Kit or AllPrep^TM^ DNA/RNA Micro Kit purchased from Qiagen (Valencia, CA, USA) according to the manufacturer’s instructions. To analyze global genomic DNA methylation, 500 ng of genomic DNA was subjected to the Luminometric Methylation Assay (LUMA) [37,38], with a standard curve derived from the in vitro differential methylated lambda phage DNA (New England BioLabs, Ipswich, MA, USA). This method is based on combined DNA cleavage by methylation-sensitive restriction enzymes (HpaII and MspI, New England BioLabs, Ipswich, MA, USA) and polymerase extension assay by pyrosequencing. DNA methylation is defined as the ratio of ratio [(HpaII/EcoRI)/(MspI/EcoRI)] in each sample. If DNA is completely unmethylated, the ratio is 1.0; if DNA is 100% methylated, the ratio is zero.

To quantitatively measure DNA methylation of opsin genes, 200–500 ng of genomic DNA was bisulfite converted using EZ DNA Methylation-Gold^TM^ Kit (Zymo Research, Irvine, CA, USA) according to the manufacturer’s instructions. Following bisulfite treatment, unmethylated cytosines are converted to uracil, whereas methylated cytosines remain cytosine. PCR amplicon and sequencing primers are shown in Table S1. PCR reaction conditions were 1× HotStar Taq PCR buffer supplemented with 2.5 mmol/L MgCl2, 200 µmol/L dNTPs, and 0.75 U of HotStar Taq DNA polymerase (Qiagen, Germantown, MD, USA) in a 30 µL reaction volume. The PCR program consisted of a denaturing step of 15 min at 95 °C followed by 44 cycles of 30 s at 94 °C, 30 s at 60 °C, and 1 min at 72 °C, with a final extension of 5 min at 72 °C. Pyrosequencing was performed on a PyroMark Q24 platform (Qiagen, Germantown, MD, USA) using the PyroMark Gold Q24 reagents according to the manufacturer’s instructions. Serial pyrosequencing, stripping the template strand for subsequent annealing of a new sequencing primer, was performed when multiple sequencing primers were needed to cover the CpG sites within a single PCR product [39,40]. Pyrosequencing assays were designed using the PyroMark Assay Design Software. Pyrograms were generated and raw data were analyzed with the PyroMark Q24 software. Sequence peaks surrounding the target CpG site served as reference peaks for normalization and quality control. DNA methylation was defined as the percentage of the C peak to the C plus T peak for each CpG site.

### 4.5. Real-Time Reverse Transcription-Polymerase Chain Reaction

Total RNA was isolated from the cell pellets or retinal explants using either the RNeasy Mini Kit or AllPrep^TM^ DNA/RNA Micro Kit purchased from Qiagen according to the manufacturer’s instructions. Then, 300 ng to 1µg of total RNA were used to generate cDNA using iScript cDNA Synthesis Kit (Bio-Rad, Hercules, CA, USA), and 1 µL cDNA was used in PCR in iQ SYBR Green Supermix (Bio-Rad, Hercules, CA, USA), with the following cycles: 95 °C for 3 min followed by 50 cycles at 95 °C for 10 s, at 63 °C for 30 s, and 72 °C for 30 s. The cycle threshold number (Ct) was determined for each PCR using either IQ5 Real-Time PCR Detection System (Bio-Rad) or CFX96 TouchTM Real-Time PCR Detection System (Bio-Rad, Hercules, CA, USA). Relative expression was calculated with the Pfaffl method [41] normalized to *HPRT1*, *HMBS* and/or *GAPDH*. An experiment consisted of triplicate amplification reactions for each gene product being analyzed. Results were expressed as the relative abundance of a specific mRNA between the control and experimental sample (fold change, mean ± SD). Sequences and product sizes are shown in Table S2.

### 4.6. Immunoblot Analysis

Total protein was extracted from cell pellets by lysing in RIPA buffer (Sigma-Aldrich, Saint Louis, MO, USA) containing 1x protease inhibitor cocktail (Roche Diagnostics, Indianapolis, IN, USA). EZQ Protein Quantitation Kit (Molecular Probes, Eugene, OR, USA) was used to measure protein concentration. Forty micrograms of total protein were denatured and electrophoresed on Any kD^TM^ Mini-Protean TGX precast polyacrylamide gels (Bio-Rad, Hercules, CA, USA), electroblotted on nitrocellulose membranes (Bio-Rad, Hercules, CA, USA), and probed with the respective antibodies against acetyl-histone H3 (Lys9; C5B11; 1:1000)) or β-actin (D6A8; 1:1000) purchased from Cell Signaling Technology (Danvers, MA, USA). The bound antibodies were visualized with horseradish peroxidase-linked anti-rabbit IgG (1:3000; Cell Signaling Technology, Danvers, MA, USA) and enhanced chemiluminescence (Amersham, Pittsburgh, PA, USA). β-actin in the corresponding cell lysates was used as an additional loading control.

### 4.7. Statistical Analysis

All statistical analyses were performed using GraphPad Prism 6.0 (San Diego, CA, USA) or Microsoft Excel software. Data were subjected to ANOVA, Tukey’s multiple comparisons test, or Student’s *t*-test (one-way). Differences with *p* < 0.05 were considered statistically significant.

## 5. Conclusions

In terminally differentiated human retinoblastoma cells in culture, we found that DNMTi and HDACi, separately and in combination, upregulated the expression of opsin genes. In contrast, postnatal murine retinal explants cultured ex vivo with 5-Aza-dc showed decreased *Rho* expression and shifted the *Rho* expression time-course curve to an earlier time point. While epigenetics plays a role in the expression of opsin genes, the differing drug effects observed indicate that the roles of these epigenetic modifications differ in the adult and developing retina.

## Data Availability

Data sharing not applicable.

## Supplementary Materials

The following are available online at https://www.mdpi.com/article/10.3390/ijms23031408/s1.

## Abbreviations

DNMT	DNA methyltransferase
5-Aza-dc	5-aza-2’-deoxycytidine
HDACi	histone deacetylase inhibitor
SB	sodium butyrate
SAHA	suberoylanilide hydroxamic acid

## References

[1] Klose R.J., Bird A.P. (2006). Genomic DNA methylation: The mark and its mediators. Trends Biochem. Sci..

[2] Antequera F., Bird A. (1993). Number of CpG islands and genes in human and mouse. Proc. Natl. Acad. Sci. USA.

[3] Otteson D.C. (2011). Eyes on DNA methylation: Current evidence for DNA methylation in ocular development and disease. J. Ocul. Biol. Dis. Inform..

[4] Song F., Mahmood S., Ghosh S., Liang P., Smiraglia D.J., Nagase H., Held W.A. (2009). Tissue specific differentially methylated regions (TDMR): Changes in DNA methylation during development. Genomics.

[5] Cedar H., Bergman Y. (2009). Linking DNA methylation and histone modification: Patterns and paradigms. Nat. Rev. Genet..

[6] Bestor T.H. (2000). The DNA methyltransferases of mammals. Hum. Mol. Genet..

[7] Goffin J., Eisenhauer E. (2002). DNA methyltransferase inhibitors-state of the art. Ann. Oncol. Off. J. Eur. Soc. Med. Oncol./ESMO.

[8] Yoo C.B., Jones P.A. (2006). Epigenetic therapy of cancer: Past, present and future. Nat. Rev. Drug Discov..

[9] Santi D.V., Norment A., Garrett C.E. (1984). Covalent bond formation between a DNA-cytosine methyltransferase and DNA containing 5-azacytosine. Proc. Natl. Acad. Sci. USA.

[10] Haberland M., Montgomery R.L., Olson E.N. (2009). The many roles of histone deacetylases in development and physiology: Implications for disease and therapy. Nat. Rev. Genet..

[11] Kelly W.K., Marks P.A. (2005). Drug insight: Histone deacetylase inhibitors--development of the new targeted anticancer agent suberoylanilide hydroxamic acid. Nat. Clin. Pract. Oncol..

[12] Lin H.Y., Chen C.S., Lin S.P., Weng J.R., Chen C.S. (2006). Targeting histone deacetylase in cancer therapy. Med. Res. Rev..

[13] Marks P.A., Richon V.M., Miller T., Kelly W.K. (2004). Histone deacetylase inhibitors. Adv. Cancer Res..

[14] Peart M.J., Smyth G.K., van Laar R.K., Bowtell D.D., Richon V.M., Marks P.A., Holloway A.J., Johnstone R.W. (2005). Identification and functional significance of genes regulated by structurally different histone deacetylase inhibitors. Proc. Natl. Acad. Sci. USA.

[15] Cameron E.E., Bachman K.E., Myohanen S., Herman J.G., Baylin S.B. (1999). Synergy of demethylation and histone deacetylase inhibition in the re-expression of genes silenced in cancer. Nat. Genet..

[16] Chai G., Li L., Zhou W., Wu L., Zhao Y., Wang D., Lu S., Yu Y., Wang H., McNutt M.A. (2008). HDAC inhibitors act with 5-aza-2’-deoxycytidine to inhibit cell proliferation by suppressing removal of incorporated abases in lung cancer cells. PLoS ONE.

[17] Esteller M. (2007). Cancer epigenomics: DNA methylomes and histone-modification maps. Nat. Rev. Genet..

[18] Jones P.A., Baylin S.B. (2002). The fundamental role of epigenetic events in cancer. Nat. Rev. Genet..

[19] Peng G.H., Chen S. (2011). Active opsin loci adopt intrachromosomal loops that depend on the photoreceptor transcription factor network. Proc. Natl. Acad. Sci. USA.

[20] Rao R.C., Hennig A.K., Malik M.T., Chen D.F., Chen S. (2011). Epigenetic regulation of retinal development and disease. J. Ocul. Biol. Dis. Inform..

[21] Deeb S.S., Bisset D., Fu L. (2010). Epigenetic control of expression of the human L- and M-pigment genes. Ophthalmic Physiol. Opt. J. Br. Coll. Ophthalmic Opt..

[22] Merbs S.L., Khan M.A., Hackler L., Oliver V.F., Wan J., Qian J., Zack D.J. (2012). Cell-specific DNA methylation patterns of retina-specific genes. PLoS ONE.

[23] Chen B., Cepko C.L. (2007). Requirement of histone deacetylase activity for the expression of critical photoreceptor genes. BMC Dev. Biol..

[24] Rhee K.D., Yu J., Zhao C.Y., Fan G., Yang X.J. (2012). Dnmt1-dependent DNA methylation is essential for photoreceptor terminal differentiation and retinal neuron survival. Cell Death Dis..

[25] Chen S., Wang Q.L., Nie Z., Sun H., Lennon G., Copeland N.G., Gilbert D.J., Jenkins N.A., Zack D.J. (1997). Crx, a novel Otx-like paired-homeodomain protein, binds to and transactivates photoreceptor cell-specific genes. Neuron.

[26] Bandyopadhyay M., Rohrer B. (2010). Photoreceptor structure and function is maintained in organotypic cultures of mouse retinas. Mol. Vis..

[27] Donovan S.L., Dyer M.A. (2006). Preparation and square wave electroporation of retinal explant cultures. Nat. Protoc..

[28] Sawamiphak S., Ritter M., Acker-Palmer A. (2010). Preparation of retinal explant cultures to study ex vivo tip endothelial cell responses. Nat. Protoc..

[29] Wallace D.M., Donovan M., Cotter T.G. (2006). Histone deacetylase activity regulates apaf-1 and caspase 3 expression in the developing mouse retina. Investig. Ophthalmol. Vis. Sci..

[30] Swaroop A., Kim D., Forrest D. (2010). Transcriptional regulation of photoreceptor development and homeostasis in the mammalian retina. Nat. Rev. Neurosci..

[31] Boatright J.H., Stodulkova E., Do V.T., Padove S.A., Nguyen H.T., Borst D.E., Nickerson J.M. (2002). The effect of retinoids and butyrate on the expression of CRX and IRBP in retinoblastoma cells. Vis. Res..

[32] Karasawa Y., Okisaka S. (2004). Inhibition of histone deacetylation by butyrate induces morphological changes in Y79 retinoblastoma cells. Jpn. J. Ophthalmol..

[33] Madigan M.C., Chaudhri G., Penfold P.L., Conway R.M. (1999). Sodium butyrate modulates p53 and Bcl-2 expression in human retinoblastoma cell lines. Oncol. Res..

[34] Peng G.-H., Chen S. (2007). Crx activates opsin transcription by recruiting HAT-containing co-activators and promoting histone acetylation. Hum. Mol. Genet..

[35] Nasonkin I.O., Lazo K., Hambright D., Brooks M., Fariss R., Swaroop A. (2011). Distinct nuclear localization patterns of DNA methyltransferases in developing and mature mammalian retina. J. Comp. Neurol..

[36] Kaewkhaw R., Kaya K.D., Brooks M., Homma K., Zou J., Chaitankar V., Rao M., Swaroop A. (2015). Transcriptome Dynamics of Developing Photoreceptors in Three-Dimensional Retina Cultures Recapitulates Temporal Sequence of Human Cone and Rod Differentiation Revealing Cell Surface Markers and Gene Networks. Stem Cells.

[37] Karimi M., Johansson S., Ekstrom T.J. (2006). Using LUMA: A Luminometric-based assay for global DNA-methylation. Epigenetics Off. J. DNA Methylation Soc..

[38] Karimi M., Johansson S., Stach D., Corcoran M., Grander D., Schalling M., Bakalkin G., Lyko F., Larsson C., Ekstrom T.J. (2006). LUMA (LUminometric Methylation Assay)—A high throughput method to the analysis of genomic DNA methylation. Exp. Cell Res..

[39] Tost J., El Abdalaoui H., Gut I.G. (2006). Serial pyrosequencing for quantitative DNA methylation analysis. BioTechniques.

[40] Tost J., Gut I.G. (2007). DNA methylation analysis by pyrosequencing. Nat. Protoc..

[41] Pfaffl M.W. (2001). A new mathematical model for relative quantification in real-time RT-PCR. Nucleic Acids Res..

