# Supplementary material for: Regulation of Opsin Gene Expression by DNA Methylation and Histone Acetylation
Source: Int J Mol Sci. 2022 Jan 26;23(3):1408. doi: 10.3390/ijms23031408 (PMC8836077; doi:10.3390/ijms23031408)
Supplement: Supplementary file 1 [file ijms-23-01408-s001.zip › ijms-1558265-supplementary.pdf]

Table S1. Human and Mouse PCR and Pyrosequencing Primers.

Gene	Species	Primer Name	Product Size (bp)	Sequence (5'-3')	Target CpG sites Relative to TSS
RHO	Human	RHO-F1	291	GTTGTGGGTTAGTTTTGATTTAAGG	
		RHO-R1*		CCCACCCAAAATTCCCAAATC	
		RHO-S1		TTTTGGTTATGAGGGTTT	-1893, -1859, -1834, -1822, -1803
		RHO-F2	258	AGGGGTTTGTAAATAAATGTTTAATGA	
		RHO-R2*		ACTTTCTAATTTATTCTCCCAATCTCT	
		RHO-S2		AGGTTATGTGTTTGGTA	-480
		RHO-S3		ATTGGATGATTTTAGAGGT	-363
		RHO-F3	241	TTGAGTTGGGATTTTGGGATAGATAAG	
		RHO-R3*		TATAAAATAACCTCCCCCTCCT	
		RHO-S4		TTTGGTTTTTTTTAGAAGTTAATTA	-106
		RHO-F4	273	AGGAAGTTGATGGGGAAGT	
		RHO-R4*		CAAAACCCAAAATCATCCAACTAA	
		RHO-S5		TGGTTTTTGTGGTTGA	70
		RHO-S6		AGGTAGTATTGTGGG	124, 141, 144, 155, 166
		RHO-S7		GTTGATGGGGAAGTTT	217, 238
		RHO-F5	361	TGATTGAATATATGAGGGTTTTGGATAA	
		RHO-R5*		ACTAAACTTCCCCATCAACTT	
		RHO-S8		TGAAGTTATTTAGGATTATGAAG	274, 280, 303, 334.
		RHO-S9		AAGAAGTTTTTTAAATTGTATTTTG	403, 409
OPN1LW	Human	OPN1LW-F1	191	GGTGGGTGGTGAAATTGTTT	
		OPN1LW-R1*		AATCAATCTACCCCCCCTAC	
		OPN1LW-S1		GAAAGTTTAGGGAGATTATT	-485, -473, -469, -457, -441, -428, -415
		OPN1LW-F2	284	GGGTTTTTAAGAGAATTATATGAGAAAGG	
		OPN1LW-R2*		CCTCTTACTCCCCTACTCCTA	
		OPN1LW-S2		GAGAATTATATGAGAAAGGAG	-360, -310, -287, -283
		OPN1LW-S3		AGAATATATAAATATAGAGAGGGT	-231, -215, -201, -186
		OPN1LW-S4		AGTTTGGTTTTTAGTAAATTTTT	-151, -143, -137
		OPN1LW-F3	276	TTTTAGGAGTAGGGGAGTAAGA	
		OPN1LW-R3*		ATACTAAACTAAATACTATCCTCATAAC	
		OPN1LW-S5		GGTTTAGTAGTGGAGTT	90, 97, 104
Rho	Mouse	Rho-F6	169	AGAGGATTTTGGGGTAGATAAG	
		Rho-R6*		TCCCTAAACCAAAAACTAATTCAACA	
		Rho-S10		ATTTTTTTTTTTTTTTATTTAAGGG	-166
		Rho-S11		ATTTTGGTTTTTTTGTAAGTTAAT	-128, -107, -91, -89

TSS, transcription start site. *, biotinylated primer.

Table S2. Human and Mouse qPCR Primers.

Gene	Species	Primer Name	Product Size (bp)	Sequence (5'-3')
RHO	Human	RHO-F1	88	TCATCTATATCATGATGAACAAGCAG
		RHO-R1		GCCTCATCGTCACCCAGT
OPN1LW	Human	OPN1LW-F1	73	CGCTATCATCATGCTCTGCT
		OPN1LW-R1		AGACTCTTTCTGCTGCTTTGC
GAPDH	Human	GAPDH-F1	109	CTGACTTCAACAGCGACACC
		GAPDH-R1		TAGCCAAATTCGTTGTCATACC
HPRT1	Human	HPRT1-F1	132	GACCAGTCAACAGGGGACAT
		HPRT1-R1		CCTGACCAAGGAAAGCAAAG
HMBS	Human	HMBS-F1	215	CTCTGCGGAGACCAGGAGT
		HMBS-R1		GGTACCCACGCGAATCAC
Rho	Mouse	Rho-F2	193	TCACGCTATCATGGGTGTGGTCTT
		Rho-R2		AGGAATGGTGAAGTGGACCACGAA
Opn1mw	Mouse	Opn1mw-F1	140	ACTCAGCATCATCGTGCTCTGCTA
		Opn1mw-R1		AGTATGCGAAGACCATCACCACCA
Opn1sw	Mouse	Opn1sw-F1	125	CTTTGGTCGCCATGTTTGTGCTCT
		Opn1sw-R1		TGCTGCCGAAGGGTTTACAGATGA
Cyclophilin A	Mouse	Cyclophilin A-F1	133	CAGACGCCACTGTCGCTTT
		Cyclophilin A-R1		TGTCTTTGGAACTTTGTCTGCAA
